# Helicobacter pylori infection and risk for developing dementia: an evidence-based meta-analysis of case-control and cohort studies

**DOI:** 10.18632/aging.203571

**Published:** 2021-09-24

**Authors:** Nan-Yang Liu, Jia-Hui Sun, Xue-Fan Jiang, Hao Li

**Affiliations:** 1 Xiyuan Hospital, China Academy of Chinese Medical Sciences, Beijing, China; 2 Beijing University of Traditional Chinese Medicine, Beijing, China; 3 Wangjing Hospital, China Academy of Chinese Medical Sciences, Beijing, China

**Keywords:** helicobacter pylori, dementia, Alzheimer’s disease, systematic review and meta-analysis

## Abstract

Background: Infection with multiple pathogens may play a key role in the pathogenesis of dementia. Whether Helicobacter pylori (*H. pylori*) infection is associated causally with dementia is controversial.

Objective: We conduct a meta-analysis of case-control and cohort studies on the association between *H. pylori* infection and the risk for all-cause and Alzheimer’s disease (AD) dementia.

Methods: Two independent reviewers searched the PubMed, Cochrane Library, and Embase databases with English language restrictions from the date of conception to September 18, 2020. The primary analysis was as follows: the exposure variable was *H. pylori* infection, and the outcome was incident all-cause and AD dementia. Pooled odds ratios (OR), relative risk (RR), and corresponding 95% confidence intervals (CI) were obtained using the fixed-or random-effect model. Forest plots were generated to summarize the results.

Results: Ten studies involving 96,561 participants were included in the meta-analysis: 5 case-control studies and 5 cohort studies. The overall pooled cohort studies showed a significant positive association between *H. pylori* infection and all-cause dementia with pooled RR of 1.36 (95% CI, 1.11-1.67). There was no association between *H. pylori* infection and risk for developing AD: RR of 1.33 (95% CI, 0.86-2.05) in cohort studies, and OR of 1.72 (95% CI, 0.97-3.04) in case-control studies. Significant heterogeneity was showed in each comparison group.

Conclusion: This meta-analysis supports a positive association between *H. pylori* infection and the risk of all-cause dementia, but not AD dementia. Due to the interference of confounding factors, randomized controlled trials are needed to prove their causality.

## INTRODUCTION

Dementia is a serious social and medical problem that affects the health of people older than 65 years worldwide. The most common types of dementia were Alzheimer’s disease (AD) and vascular dementia, accounting for 60%-70% and 30%, respectively. Currently, there are more than 50 million people with dementia worldwide, and this number will increase to 152 million by 2050 [1]. The 2019 Alzheimer’s disease facts and figures in the United States showed that between 2000 and 2017, deaths resulting from stroke, heart disease, and prostate cancer decreased, whereas reported deaths from AD increased 145% [1]. Such a situation is not optimistic in Asia. Recent data show that the number of dementia in China accounts for approximately 25% of the total number of dementia in the world [2].

The identified risk factors for dementia include sociodemographic structure (age, gender, low education level) and family history. In addition, infections, diabetes, hypertension, and stroke can also contribute to the occurrence of dementia [3]. Emerging data have demonstrated that infection with several important pathogens may be a hazard factor for cognitive impairment, dementia, and AD in particular [4]. Among them, Helicobacter pylori (*H. pylori*) is the most interesting for researchers [5, 6]. *H. pylori*, the only microbial species currently known to survive in the human stomach, was successfully isolated from the gastric mucosa of patients with chronic active gastritis for the first time in 1984 [7]. Infection occurs mainly in childhood and generally survives in the body. However, most people are asymptomatic during infection [8]. Chronic *H. pylori* infection is a direct inducement of chronic gastritis, peptic ulcers, and gastric cancer. Interestingly, *H. pylori* has also been identified as a risk factor for non-gastrointestinal diseases, such as neurodegenerative diseases, including all-cause and AD dementia [9–11].

Previous evidence suggests *H. pylori* infection as a driver of cognitive decline. The potential link between *H. pylori* infection and dementia have been investigated by several population-based case-control and cohort studies, but the results were inconsistent. Although a previous meta-analysis that combined several observational studies have reported a statistically significant association between *H. pylori* infection and all-cause dementia, the evidence was limited to inappropriate statistical methods [12]. And one of the studies even included patients with mild cognitive impairment rather than the dementia, leading to unconvincing evidence. Therefore, the purpose of this study was to conduct a systematic review and meta-analysis of case-control and cohort studies to understand the association between *H. pylori* infection and the risk of developing all-cause and AD dementia.

## RESULTS

### Literature search

**Figure 1:**
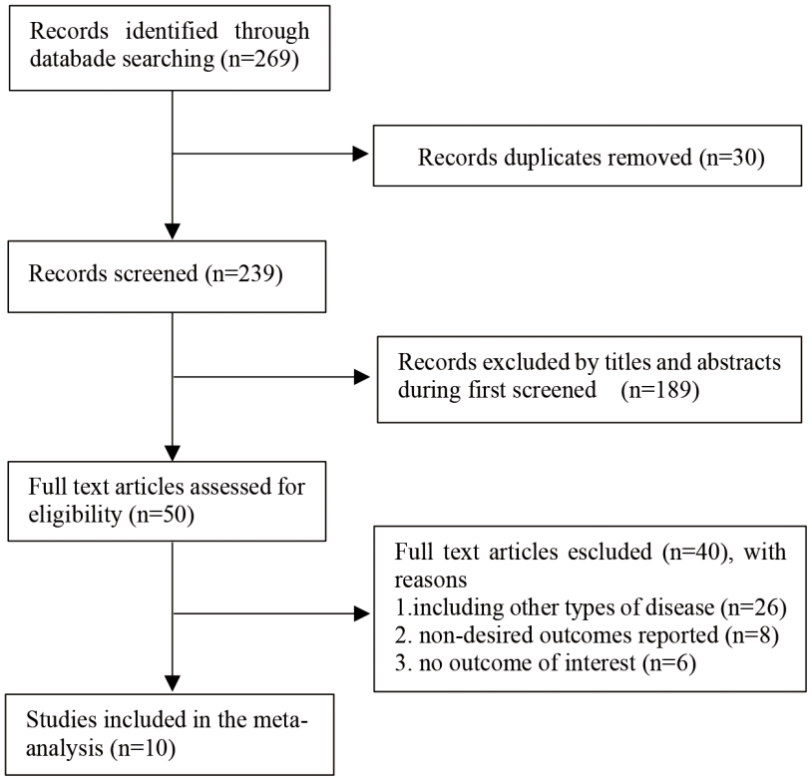
Flow diagram of the literature selection process.

A total of 269 records were identified from all databases. After excluding 30 duplicate analysis, 239 records were filtered by reading the title and abstract. A total of 189 items were excluded due to irrelevant topics. We screened the full text of the remaining 50 studies and identified 10 studies that met the inclusion criteria of the meta-analysis, including 5 case-control studies and 5 cohort studies. The detailed PRISMA flowchart describing the literature search process is presented in Figure 1.

### Study characteristics and quality

The ten studies, published between 2003 and 2018, varied in design, study population, sample size, outcome variables, and methods used to determine H. pylori infection. With respect to the countries that conducted these studies, one of them was conducted in the United States [13], six in Europe [14–19], two in China [20, 21], and one in Japan [22]. The sample sizes of the studies ranged from 61 to 83965. Of five case-control studies, four studies used nondementia control groups [16, 19, 20, 22], and one study used an iron deficiency anemic control group [18]. Nine studies reported AD [13–16, 18–22], whereas 6 reported all-cause dementia [13–17, 21]. The follow-up time for the cohort study ranged from 3 to 20 years. The average age of the study samples in each group was between 59.3 and 78.5 years old. The identification of *H. pylori* infection involved serum IgG antibodies, rapid urine tests, and gastric mucosal histology. Six studies detected IgG antibodies in the serum [13–15, 17, 19, 20], and one study measured histology together with serum IgG antibodies [16]. One study was based on codes in the International Classification of Diseases 9th edition from national registries [21], one used histology [18] and one used rapid urine test [22]. Two of ten studies did not adjust for confounding factors [14, 16]. In the Newcastle-Ottawa scale assessment, all studies received a high score of ≥ 6 stars, indicating that the quality of the literature was reliable (Table 1).

**Table 1:** Characteristics of case–control and cohort studies included in the meta-analysis.

**Study**	**Year/location**	**Population/age M(SD)**	**Duration (yrs)**	***H. pylori* detection**	**Outcome**
**Cohort studies**					
Baudron [17]	2013, France	N=603 *H. pylori* positive 73.1(6.4) *H. pylori* negative 74.3(6.5)	20	Serum *H. pylori* IgG antibodies	all-cause dementia
Huang [21]	2014, Chian	N=83965 *H. pylori* positive 63.2 (13.5) *H. pylori* negative 63.6 (13.4)	12	*H. pylori* infection (ICD-9 code 041.86)	all-cause dementia; AD; non-AD
Fani [15]	2018, Netherlands	N=4275 *H. pylori* positive 69.3 (8.6) *H. pylori* negative 67.5 (8.5)	10.4	Serum *H. pylori* IgG antibodies	all-cause dementia; AD
Beydoun [13]	2018, United States	N=5927 *H. pylori* positive 59.3 (0.2) *H. pylori* negative 62.8 (0.4)	3	Serum *H. pylori* IgG antibodies	all-cause dementia; AD
Von [14]	2018, France	N=689, Age≥65	10	Serum *H. pylori* IgG antibodies	all-cause dementia;AD
**Case control studies**					
Nagga [19]	2003, Sweden	N=216, Age≥60	NA	Serum *H. pylori* IgG antibodies	AD, VaD
Kountouras [18]	2006, Greece	N=80 AD 65.0(6.9) Control 62.2(8.6)	NA	histologic analysis	AD
Shiota [22]	2011, Japan	N=482 AD74.4(10.4) Control 78.5 (6.4)	NA	rapid urine test	AD
Bu [20]	2014, Chian	N=263 AD 69(9) Control 70(10)	NA	Serum *H. pylori* IgG antibodies	AD
Tsolaki [16]	2015, Greece	N=61 AD 61.34(6.526) Control 62.41(4.49)	NA	Serum *H. pylori* IgG antibodies and histological	all-cause dementia; AD; PD; LB; FTD

**Study**	**Diagnostic criteria**	**OR/HR, 95%CI**	**Adjusting for confounders**	**NOS**
Baudron [17]	NINCDS-AD-RDA	Dementia: HR 1.46 (95% CI 1.01–2.11)	Age, sex, education APOE4, basel+H4:H18ine MMSE score, obesity, CVD	6
Huang [21]	ICD-9-CM	Dementia: HR 1.51, 95%CI 1.25–1.82 AD: HR 0.66, 95%CI 0.30–1.46 Non-AD: HR 1.60, 95%CI 1.32–1.95	Age, sex, comorbidities hypertension, DM, depression, head injury	6
Fani [15]	DSM-III-R, NINCDS-AD-RDA	Dementia: HR 1.03, 95%CI 0.86–1.22 AD: HR 1.06 95%CI 0.87–1.29	Adjusted for age, sex, study cohort, education, smoking, systolic and diastolic blood pressure, anti-hypertensive drug use, body mass index (BMI), cholesterol, high-density lipoprotein (HDL) cholesterol, triglycerides, APOEε carrier status, stroke, diabetes mellitus, ethnicity, and serum lipid–reducing agents.	7
Beydoun [13]	NA	Dementia: HR1.44, 95%CI 1.05–1.98 AD: HR 1.45 (95%CI 1.03–2.04)	Adjusted for education, poverty income ratio, smoking, weight ststus, hypertension, diabetes, and dyslipidemia	7
Von [14]	NA	Dementia: HR 1.70 (95%CI 1.05–2.74) AD: HR 2.85 (95% CI 1.58–5.12)	NA	8
Nagga [19]	ICD-10	*H. pylori* positivity: 55.3% in AD patients, 55.6% in AD with CVD, 49.2% in VaD, 62.5% in the cognitively impaired group, 50.5% in the controls	Age	6
Kountouras [18]	NINCDS-AD-AD-RDA	OR 8.4 (95% CI 2.4–28.7)	The groups were comparable regarding SES	7
Shiota [22]	NINCDS–AD-RDA	OR 0.94 (95% CI 0.56–1.58)	Age, sex	8
Bu [20]	NINCDS–AD-RDA	OR 1.39 (95% CI 0.80–2.40)	Age, sex, education, APOE, genotype, CHD, DM, hypercholesterolemia, hypertension	6
Tsolaki [16]	MMSE, FRSSD, NI	68.33% in patients with dementia 45.16% with control	NA	6

AD, Alzheimer’s Disease; DSM-III-R, Statistical Manual of Mental Disorders–3rd Edition Revised; FRSSD, Functional Rating Scale for Symptoms of Delirium; ICD-9-CM, International Classification of Diseases 9, Ninth Revision, Clinical Modification; ICD-10, International Classification of Diseases 10; MMSE, Mini Mental State Examination; M(SD), Mean (standard deviation); N, Number of population; NA, not available; NINCDS–ADRDA, National Institute of Neurological Disorders and Stroke and Alzheimer’s Disease and Related Disorders Association; NI, Neuropsychiatric Inventory.

### Meta-analysis of the association between *H. pylori* infection and all-cause dementia in cohort study

**Figure 2:**
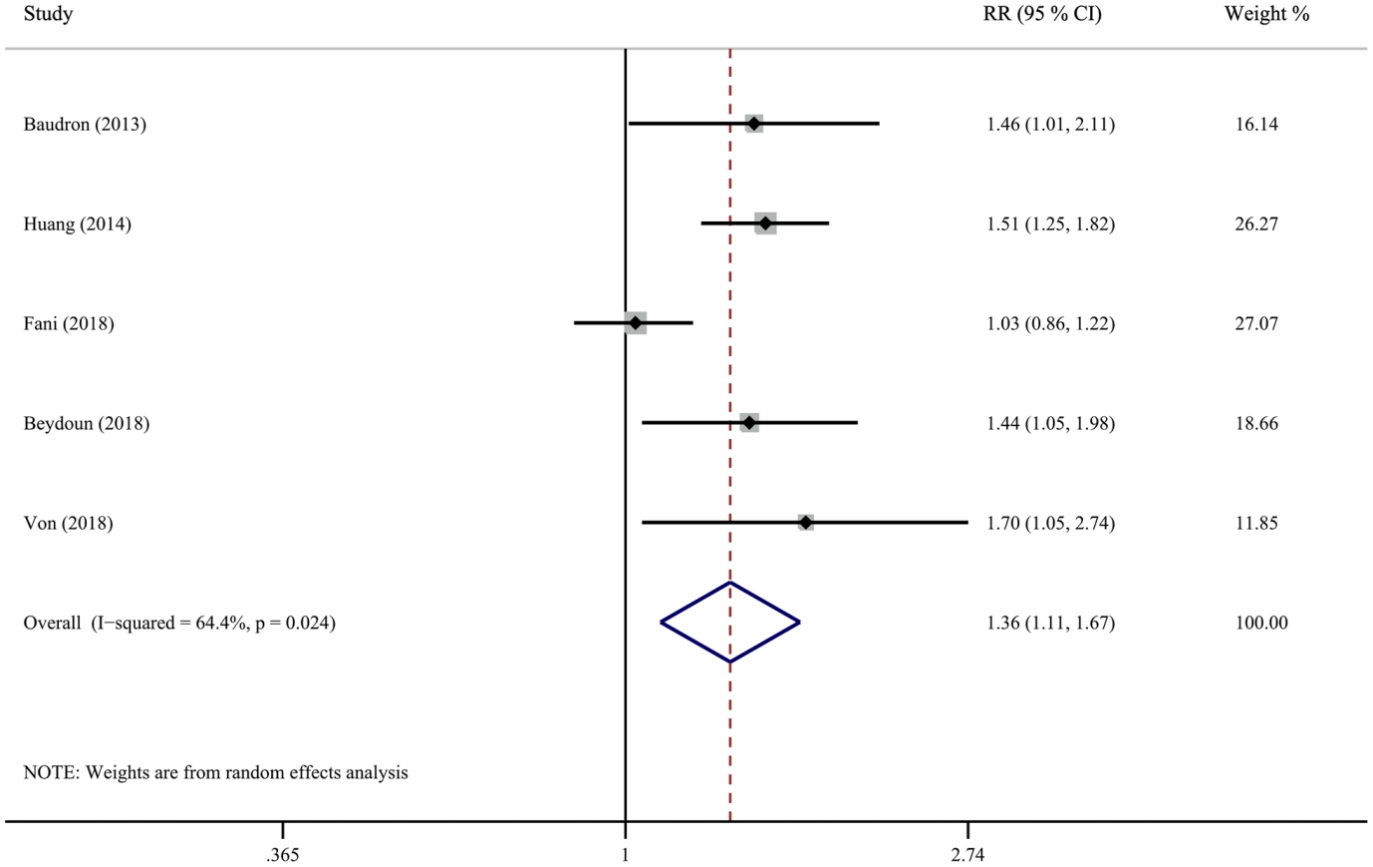
Forest plot presents the association between Helicobacter pylori infection and the risk of all-cause dementia in cohort study.

Five cohort studies investigated the correlation between *H. pylori* infection and all-cause dementia, including two retrospective and three prospective. Figure 2 shows the results of pooled RR with a random-effects model. Four studies demonstrated a significant positive association and the RR for the association ranged from 1.03 to 1.70. Overall, the pooled results indicated that *H. pylori* infection participants had a considerably risk of developing all-cause dementia compared to those negative for *H. pylori* (RR = 1.36; 95% CI = 1.11 - 1.67), with significant heterogeneity (*I*^2^ = 64.4%, *P* = 0.024). Sensitivity analysis was assessed by removing each study in sequence and re-analysing the data shows that the study of Fani et al. has an impact on the results (Supplementary Figure 1). Interestingly, the heterogeneity decreased to 0% after excluding this study, while the effect estimates increased (RR = 1.50; 95% CI = 1.31-1.73) (Supplementary Figure 2).

### Meta-analysis of the association between *H. pylori* infection and AD dementia in cohort study

**Figure 3:**
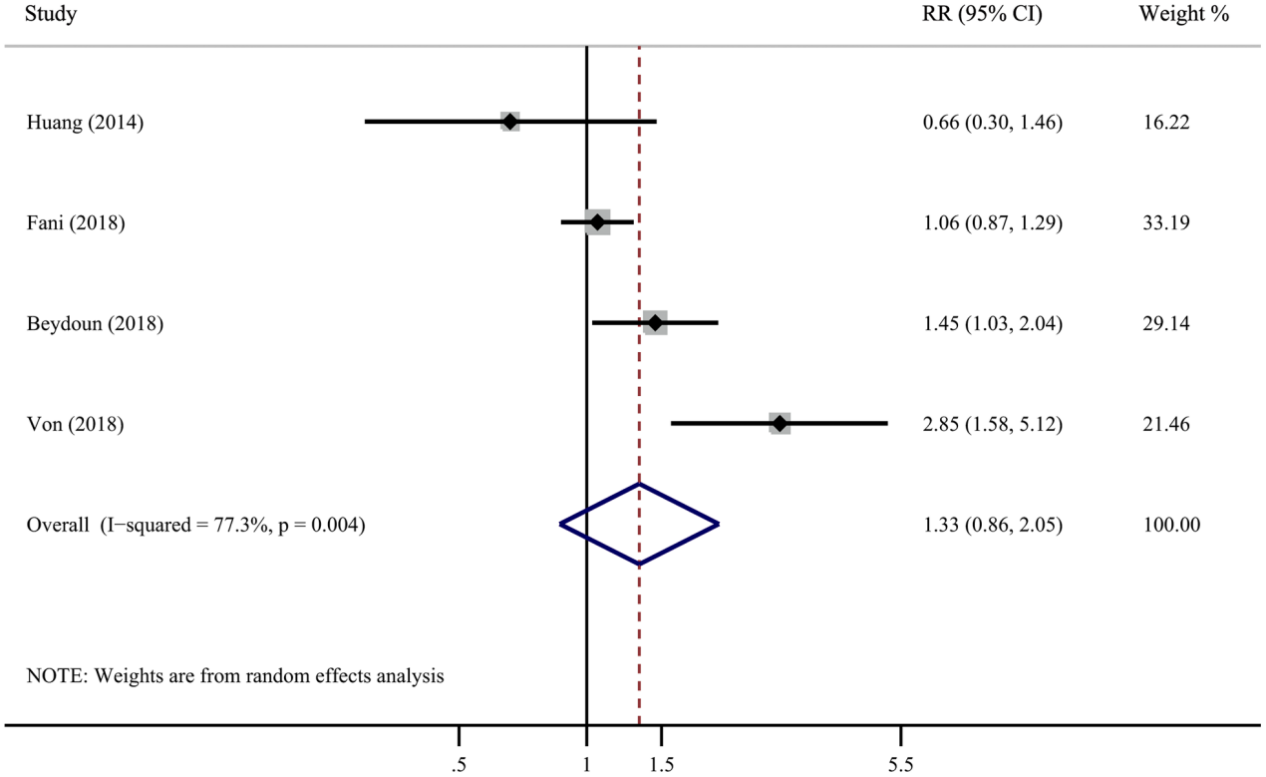
Forest plot presents the association between Helicobacter pylori infection and the risk of Alzheimer’s disease in cohort study.

Figure 3 shows the results of pooled RRs with a random-effect model of AD dementia. Four cohort studies reported the association between *H. pylori* infection and AD dementia, whereas two studies reported a significant positive association. The pooled RRs of developing AD after *H. pylori* infection in cohort studies were 1.33 (95% CI, 0.86-2.05), demonstrating that there is no causal association. Sensitivity analysis was used to assess the robustness of the results, which resulted in almost identical risk estimates. (Supplementary Figure 3).

### Meta-analysis of the association between *H. pylori* infection and AD dementia in case-control study

**Figure 4:**
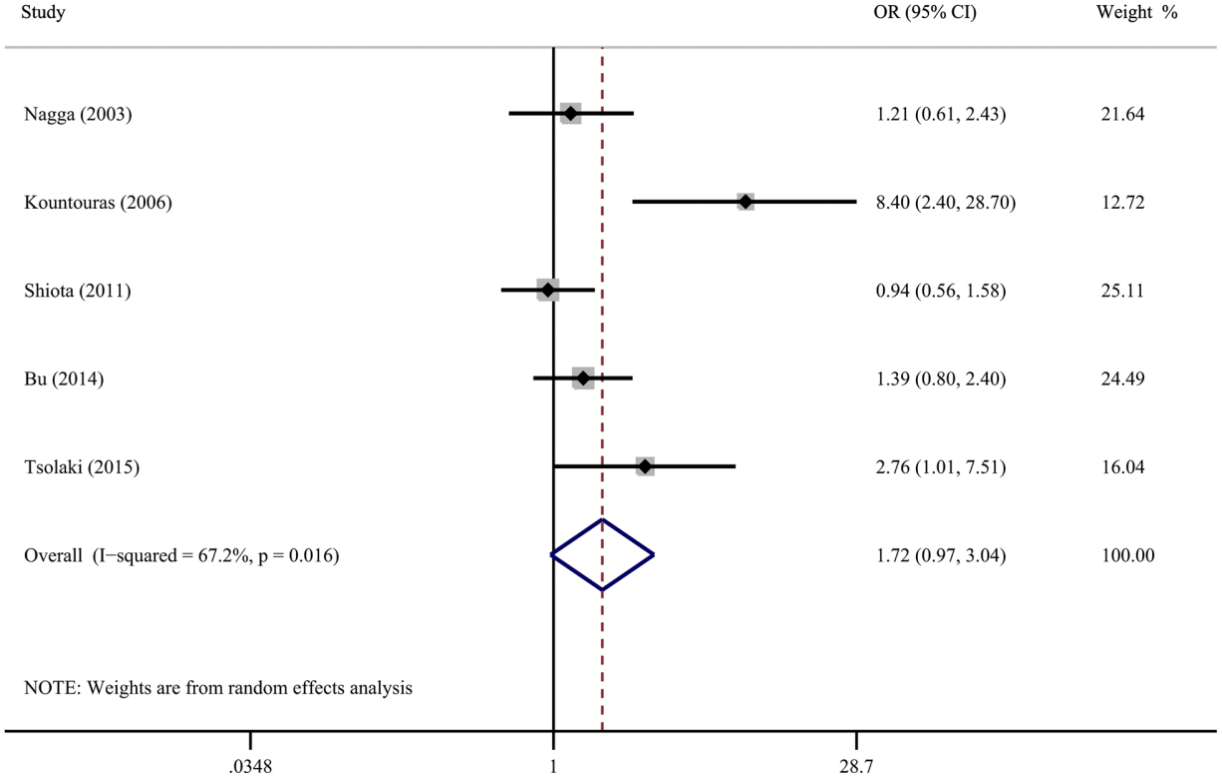
Forest plot presents the association between Helicobacter pylori infection and the risk of Alzheimer’s disease in case-control study.

Five case-control studies described the connection between *H. pylori* infection and AD dementia. The pooled results of the random-effect model demonstrate that there is no correlation between *H. pylori* infection and the incidence of AD (OR=1.72; 95% CI=0.97-3.04) (Figure 4), with significant heterogeneity (*I*^2^ = 67.2%, *P* = 0.016). Evaluating the robustness of the results by removing each study in sequence and reanalyzing the data did not lead to remarkable difference in the results (Supplementary Figure 4). However, excluding the study of Kountouras et al. reduced the heterogeneity to 19.1% (Supplementary Figure 5).

### Publication bias

The Begg rank correlation test and Egger linear regression test documented no evidence of publication bias among studies between *H. pylori* infection and the risk of all-cause and AD dementia (all *P* < 0.05).

## DISCUSSION

Previous researches have been performed to examine the association between *H. pylori* infection and various cognitive outcomes, including all-cause and AD dementia. However, the association is not fully understood [23–26]. Our results are limited to observational studies suggest that *H. pylori* infection may be an independent risk factor for all-cause dementia, but not for AD. In fact, the average follow-up time for these cohort studies is 3 to 20 years. This large time interval between the two diseases further confirms the assumption that *H. pylori* infection is an independent risk factor for all-cause dementia.

Positive correlation between *H. pylori* infection and all-cause dementia was found in the previous meta-analysis, which is consistent with our findings. However, the authors pooled different study types results in significant heterogeneity, which reduced the credibility of the evidence. In addition, they included a study diagnosed as mild cognitive impairment rather than AD or all-cause dementia. Our evidence is based on independent cohort and case-control studies showing that *H. pylori* infection can lead to subsequent all-cause dementia, which makes the evidence more credible.

Several neurological diseases related to *H. pylori* infection have been reported so far, such as AD, stroke [27], Parkinson’s disease [28] and multiple sclerosis [29]. In some disease, only the correlation is explained without a clear interpretation of the pathogenic mechanism, which makes this a very interesting and controversial topic. However, for some infected groups, the association is so powerful and the pathogenic mechanism is so obvious that guidelines for the treatment of *H. pylori* infection recommend that eradication treatment should be carried out in this case. Here, we focus on the connection among *H. pylori* infection and dementia. Multiple lines of evidence have suggested that infection with *H. pylori* is a key driver of AD [30–32]. One study reported beneficial effects on cognitive and functional status parameters of patients with AD after *H. pylori* eradication [33]. Another study showed a higher 5-year survival rate after *H. pylori* eradication in AD patients [34]. However, our study found no evidence supporting the connection between *H. pylori* infection and AD susceptibility, which is inconsistent with those reports. Inconsistent results may be limited by the ethnicity of the included population, study design, and *H. pylori* identification method.

The potential physiological mechanisms involved in *H. pylori* infection and dementia are uncertain, but several candidate mechanisms have been identified. One possible explanation is the neuroinflammation hypothesis that involves a central event triggering neurodegeneration, including AD [35, 36]. Previous researches have demonstrated that *H. pylori* infection may cause damage to the blood-brain barrier and induce neurological diseases by releasing various inflammatory mediators, for instance, cytokines and chemokines [37, 38]. *H. pylori* infection may also indirectly affect the brain by releasing multiple cytokines, for example, tumor necrosis factor-α and interleukin-6, maintaining cerebrospinal fluid inflammatory factors at a high level and thus inducing neuroinflammation [39, 40]. In another study, MNK-28 human gastric cells were incubated with *H. pylori* peptide, and activated genes were monitored [41]. The results showed that 77 genes were modulated by the *H. pylori* peptide, of which 65 are identified in the AlzBase database and contain the characteristics of AD. Additionally, a large proportion of modulated genes (30 out of 77) pertains to the inflammation pathway.

The other explanation is that chronic gastritis induced by *H. pylori* infection can cause vitamin B12 and folate malabsorption, which leads to the accumulation of folate 5-methyltetrahydrofolate and homocysteine [42–44]. Increased homocysteine can trigger endothelial atherosclerotic thrombotic disease and AD damage [45–47]. Multiple lines of evidence have confirmed this hypothesis. For example, hyperhomocysteinemia was demonstrated to be a strong independent factor in the progress of dementia [48]. Another study also revealed that homocysteine levels are associated with cardiovascular disease and dementia in the context of chronic gastritis (including *H. pylori* causality) [49]. The authors believe that vitamin B12, folate levels and suffering from atrophic gastritis are crucial determinants of homocysteine levels but are not associated with dementia. In addition, B12 deficiency resulting from by *H. pylori* infection may be related to activation of tau and β-amyloid (Aβ) deposition [50]. Recent evidence suggests that vitamin B^12^ inhibits Aβ_42_ aggregation in a concentration-dependent manner and protects amyloid-induced cytotoxicity of human neuronal cell lines [51]. An adequate supply of vitamin B12 appears to be essential to prevent cognitive decline and prevent the progression of AD [52].

It noticeable that observational studies cannot confirm causality. Despite that, our report meets the causality of some Hill criteria. First, there is a distinct time relationship in the cohort study. *H. pylori* infection preceded the occurrence of dementia in all preliminary studies. Given that *H. pylori* is acquired during childhood, the infection precedes the onset of dementia. Second, the positive associations between different studies and populations were extensively consistent. Third, as mentioned above, the hypothesis that *H. pylori* infection predisposes to dementia is biologically plausible.

### Sources of heterogeneity

Significant heterogeneity was discovered in the current systematic review and meta-analysis due to clinical and methodological diversity, such as the differences in characteristics of demographic, determination of *H. pylori* infection and adjustment of confounding factors. The study of Fani et al. likely contributed to the heterogeneity in the exploration of the relationship between *H. pylori* infection and all-cause dementia [15]. In fact, the association in this study was significantly reduced compared with that in other studies, which may be due to the use of different diagnostic criteria. The pooled RR mildly increased after excluding this study (from 1.36 to 1.50), but there is no evidence of heterogeneity observed in the remaining researches (*I*^2^=0%, *P*=0.95). Additionally, the study of Kountouras et al. may be the main cause of heterogeneity due to a very strong association (OR=8.4). One possible reason accounting for this strong association may be that patients with anemia were included as the control group. Interestingly, previous evidence has suggested that patients with pernicious anemia were protected from infection with *H. pylori* [53].

### Limitation

The potential limitations of current research warrant consideration. First, a direct causal relationship between *H. pylori* infection and AD or all-cause dementia risk cannot be determined because observational studies were included. Therefore, the results should be interpreted cautiously. Second, as a meta-analysis of observational researches published in English, publication bias may be possible. We conducted a comprehensive retrieval of the literature to eliminate publication bias as much as possible. Third, another possible restriction of the present systematic review and meta-analysis was the use of various detection methods for *H. pylori* infection. Fourth, substantial heterogeneity was found among studies. Although the main source of heterogeneity was detected through sensitivity analysis, we still cannot exclude the probability that residual confounding could influence the results. Finally, un-controlled or un-measurable risk factors in the study may produce biases. Although conventional risk factors have been adjusted in some studies, the possibility of residual confounding factors that increase the risk of dementia cannot be excluded.

### Suggestions for further study

Based on our findings, several issues need to be considered. First, is there any causal relationship between *H. pylori* infection and AD dementia? To answer this question, the interval between the two diseases and adequate control of confounding factors should be assessed. Second, what is the exact mechanism by which *H. pylori* increase the risk of all-cause dementia? Neuroinflammation and hyperhomocysteinemia may provide some ideas. Third, can the eradication of *H. pylori* prevent or delay the development of dementia? Further studies are needed, including rigorously-designed clinical trials to tackle these issues to better understand this association and provide persuasive evidence for clinical practice in dementia prevention.

## CONCLUSIONS

In conclusion, this systematic review and meta-analysis suggest that *H. pylori* infection may be associated with an increased risk of all-cause dementia, but not AD dementia. Future research on the pathogenic mechanism between the two diseases may lead to the development of novel therapies. The clinical implications lie in maintaining vigilance against dementia in elderly patients infected with *H. pylori*, and early detection and timely medical treatment for *H. pylori* patients through a multidisciplinary approach.

## MATERIALS AND METHODS

### Search strategy and study selection

This systematic review and meta-analysis were conducted based on the Meta-analysis Of Observational Studies in Epidemiology (MOOSE) [51]. Two independent reviewers (N-YL and J-HS) searched PubMed, the Cochrane Library, and Embase databases with English language restrictions from the inception to July 10, 2019. The search terms were “*Helicobacter pylori/H. pylori* or *Campylobacter pylori/C. pylori*” and at least one of the following: “dementia or Alzheimer’s disease”. Two independent researchers (N-YL and HL) evaluated the article abstracts determined by the initial search to ensure qualified study, and then obtained and evaluated all potentially relevant articles in detail. References from the latest reviews were also searched. Eligible studies were randomized controlled trials, controlled clinical trials, cohort studies, and case–control studies on the relationship between adult *H. pylori* infection and dementia. The study set the following criteria: (1) interest in exposure to *H. pylori* infection (yes or not); (2) interest in all-cause and AD dementia; (3) studies including relative risk (RR), hazard ratio (HR), or odds ratio (OR), and their corresponding 95% confidence intervals (CIs) and *P*-value (or calculated data). Intervention studies, case reports, case series, duplicate reports, letters to editors, comments, and author responses were excluded.

### Data extraction and quality assessment

The standardized data form was used to extract the following information: author name, year of publication, country, period and follow-up time, reference materials, study design and population, sample size, *H. pylori* detection method, outcome variables, and statistical adjustment of confounding factors. The RR, HR, OR, and the corresponding CIs were extracted for each study (or calculated from reported data). We used the Newcastle-ottawa scale recommended by the Cochrane Collaboration to evaluate the methodological quality of the eligible studies [52]. The evaluation content includes three parts: selection, comparability, and exposure/outcome. There are 8 items in this scale with a total score of 9. Quality assessments and data extraction were performed by two researchers (X-FJ and HL), and any discrepancies that existed were resolved by discussion.

### Statistical analysis

Stata version 12.0 (Stata Corp LP, College Station, Texas) was used to calculate the pooled ORs, HRs, RRs, and 95% CIs. Forest plots were generated to summarize the results. Heterogeneity χ^2^ test and *I*^2^ index were used to assess the heterogeneity between the eligible studies. *I*^2^≤50% were deemed to have little heterogeneity; *I*^2^>50% were considered to have considerable heterogeneity. When heterogeneity cannot readily be interpreted, we incorporate it into a random-effect model [53]. Instead, we used a fixed-effect model. Potential sources of heterogeneity were identified by sensitivity analysis. Publication bias was evaluated using the Begg rank correlation test or Egger linear regression test.

## Supplementary Material

Supplementary Figures
