# Supplementary material for: Helicobacter pylori infection and risk for developing dementia: an evidence-based meta-analysis of case-control and cohort studies
Source: Aging (Albany NY). 2021 Sep 24;13(18):22571–87. doi: 10.18632/aging.203571 (PMC8507304; doi:10.18632/aging.203571)
Supplement: Supplementary Figures [file aging-13-203571-s001.pdf]

SUPPLEMENTARY FIGURES

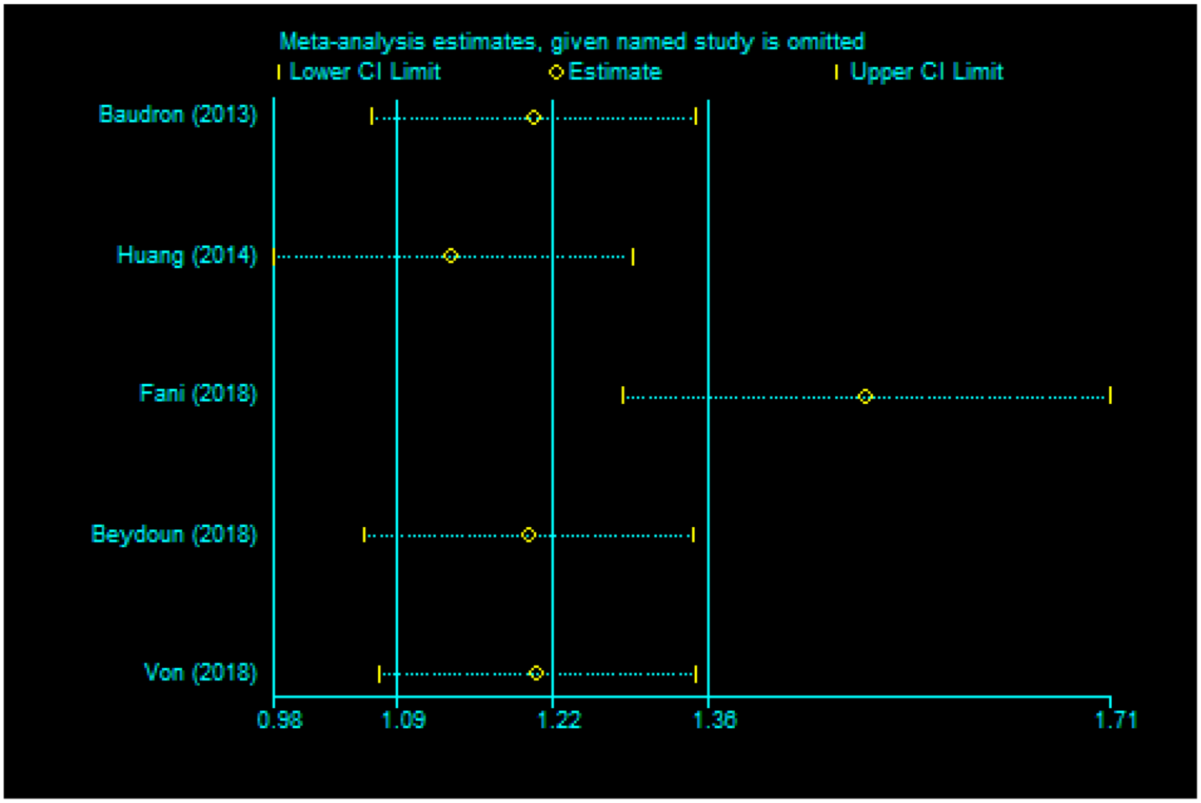

Supplementary Figure 1. Sensitivity analysis of the association between Helicobacter pylori infection and all-cause dementia in cohort study.

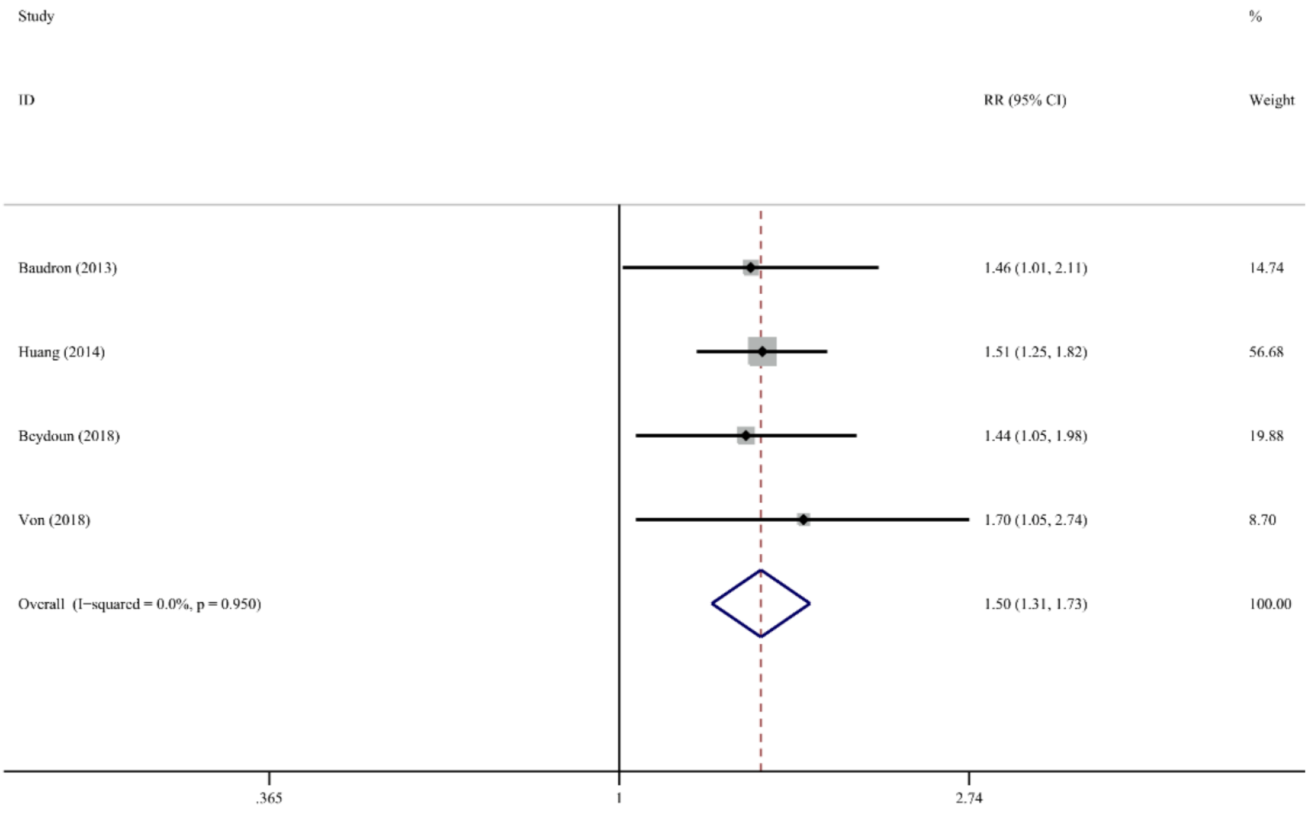

Supplementary Figure 2. Re-analysing the association between Helicobacter pylori infection and all-cause dementia after excluding the study of Fani et al.

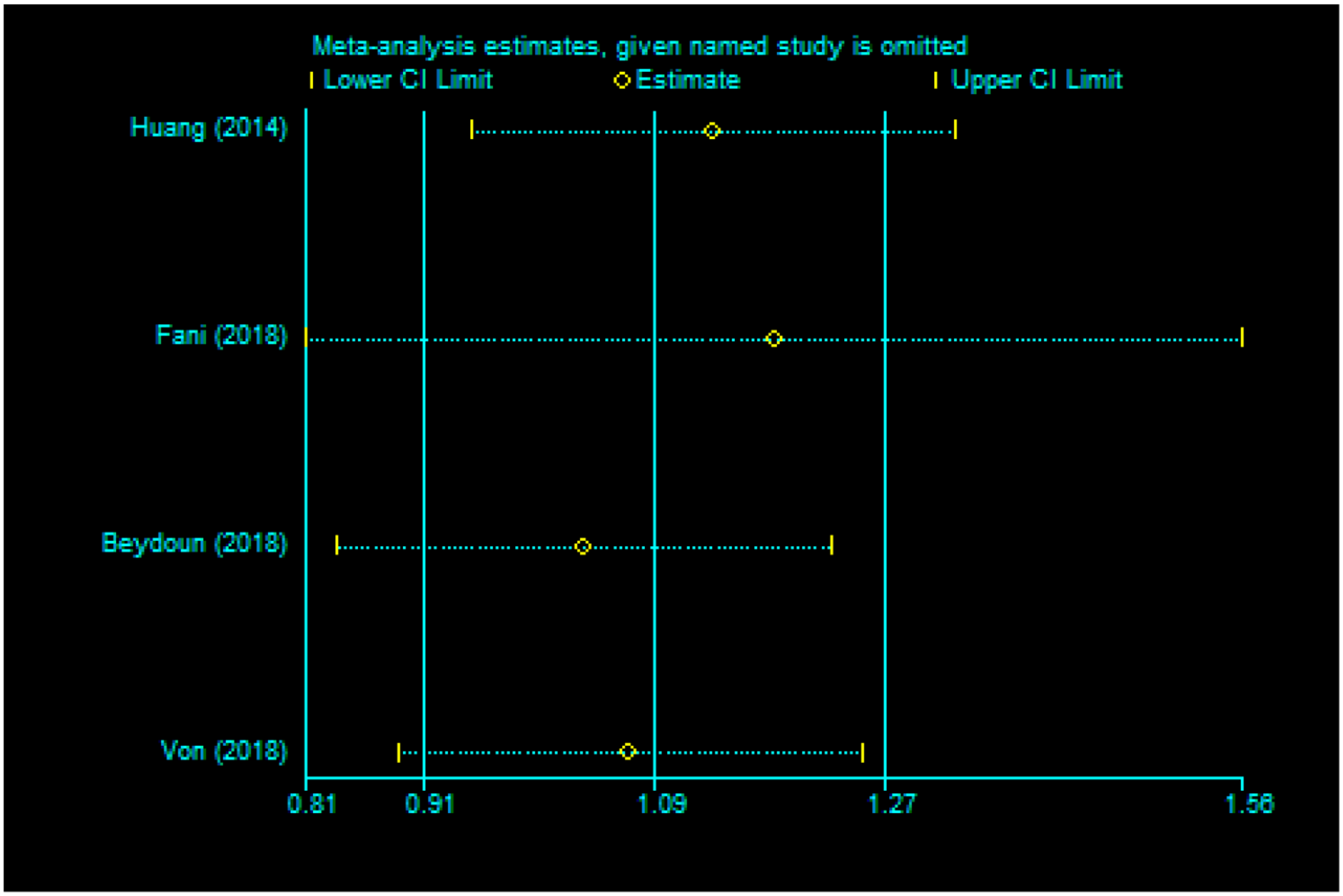

Supplementary Figure 3. Sensitivity analysis of the association between Helicobacter pylori infection and AD in cohort study.

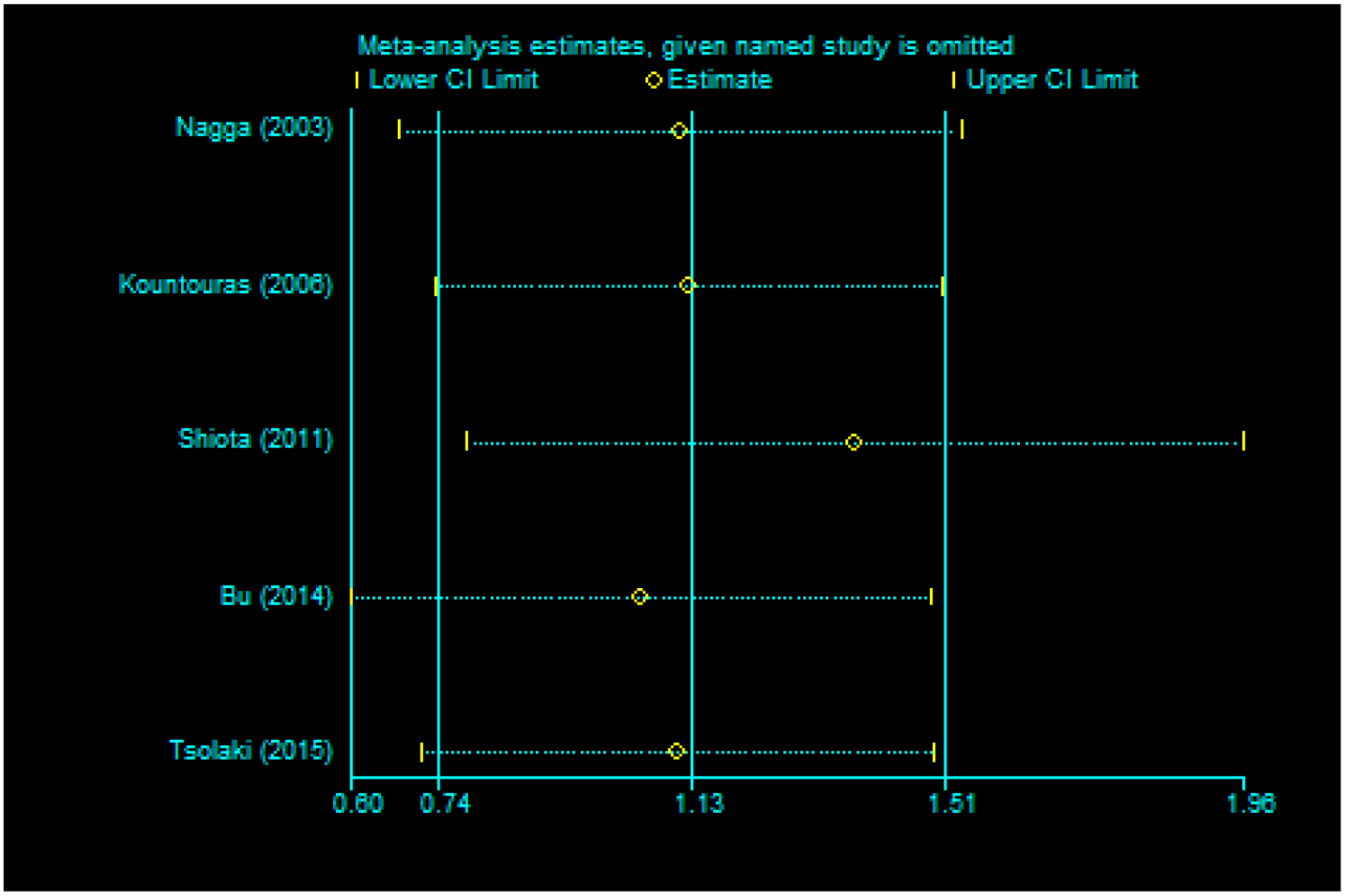

Supplementary Figure 4. Sensitivity analysis of the association between Helicobacter pylori infection and AD in case-control study.

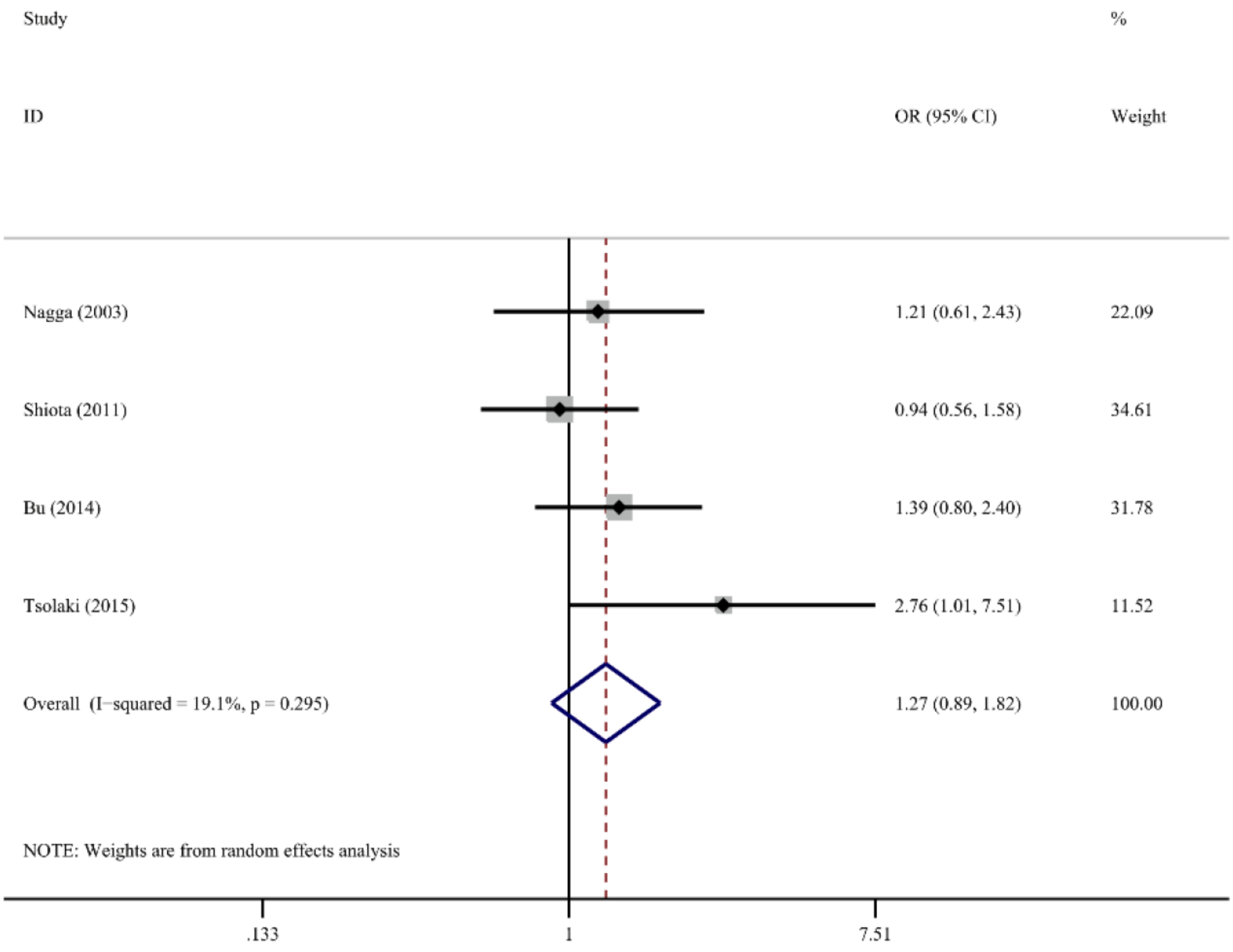

Supplementary Figure 5. Re-analysing the association between Helicobacter pylori infection and AD after excluding the study of Kountouras et al.
